# The Relationship of *CCL5* and *CCR1* Variants with Response Rate and Survival Taking into Account Thalidomide/Bortezomib Treatment in Patients with Multiple Myeloma

**DOI:** 10.3390/jcm12062384

**Published:** 2023-03-20

**Authors:** Sylwia Popek-Marciniec, Wojciech Styk, Magdalena Wojcierowska-Litwin, Aneta Szudy-Szczyrek, Paul Dudek, Grazyna Swiderska-Kolacz, Joanna Czerwik-Marcinkowska, Szymon Zmorzynski

**Affiliations:** 1 Department of Cancer Genetics with Cytogenetic Laboratory, Medical University of Lublin, 20-059 Lublin, Poland; 2 Department of Psychology, Medical University of Lublin, 20-059 Lublin, Poland; 3 Chair and Department of Hematooncology and Bone Marrow Transplantation, Medical University of Lublin, 20-059 Lublin, Poland; 4 Institute of Biology, Jan Kochanowski University, 25-406 Kielce, Poland

**Keywords:** *CCL5* gene, *CCR1* gene, chemokine receptors, chemokines, genetic variants, multiple myeloma, polymorphisms

## Abstract

(1) Background: Chemokines and chemokine receptors play an important role in tumor development. The aim of this study was to check the significance of *CCL5* and *CCR1* variants with response rate, survival, and the level of regulated on activation, normal T cells expressed and secreted (RANTES/CCL5) in multiple myeloma (MM) patients; (2) Methods: Genomic DNA from 101 newly diagnosed MM patients and 100 healthy blood donors were analyzed by Real-time PCR method (for *CCL5* and *CCR1* genotyping). In a subgroup of 70 MM patients, serum samples were collected to determine the level of RANTES; (3) Results: multivariate Cox regression showed increased risk of disease relapse or progression (HR = 4.77; *p* = 0.01) in MM patients with CG + CC genotypes of *CCL5* rs2280788. In contrast, CT + TT genotypes of *CCL5* rs2107538 were associated withdecreased risk of death (HR = 0.18; *p* = 0.028) and disease relapse or progression (HR = 0.26; *p* = 0.01). In MM patients with major genotypes of rs2280789, rs2280788, and rs2107538, higher survival rates were observed in response to treatment with thalidomide and bortezomib. Statistically significant lower RANTES levels were seen in minor genotypes and heterozygotes of *CCL5* and *CCR1* variants; (4) Conclusions: Major genotypes of *CCL5* variants may be independent positive prognostic factors in MM.

## 1. Introduction

Multiple myeloma (MM) is a hematologic malignancy, characterized by clonal expansion of plasma cells in the bone marrow (BM) [1]. MM cells proliferate and grow mainly within BM, where they create a specific environment [2]. The MM microenvironment in BM is composed of different cells, such as mesenchymal stromal cells (MSCs), dendritic cells (DCs), and T lymphocytes [3]. The immune components in the BM myeloma niche are related to MM progression and aggressiveness [4,5,6,7]. Cytokines and chemokines play an important role in regulating immune responses for cancer cells. The cytokine network is involved in the growth and progression of MM cells and also participates in the destruction of the bone marrow. The MM cells and BM microenvironment stimulate paracrine or autocrine secretion of several cytokines which may promote the growth, development, and progression of MM [8,9,10]. Chemokines are involved in the colonization and growth of myeloma cells in the bone marrow and the formation and activation of osteoclasts. The activity of osteoclasts is increased in areas close to myeloma cells, resulting in increased bone resorption and reduced bone formation [11]. Multiple myeloma cells secrete several chemokines and express a variety of chemokine receptors which participate in cell homing, tumor growth, and progression [12]. Several known chemokines show higher concentrations in MM patients’ plasma, namely: IL-1β, IL-4, IL-6, IL-8, CCL3, CCL4, and CCL5 [8,13]. 

The C-C chemokine ligand 5 (CCL5), also known as RANTES (regulated on activation, normal T cells expressed and secreted), belongs to the C-C chemokine family whose members also include CCL3 (MIP-1α) and CCL4 (MIP-1β) [14]. CCL5 is expressed by T lymphocytes, macrophages, platelets, synovial fibroblasts, tubular epithelium, and certain types of tumor cells [14]. Studies carried out so far have shown that increased levels of CCL5 in tissues or plasma are markers of an unfavorable prognosis in patients with colorectal, gastric, breast, and ovarian cancer [15,16,17,18,19]. Abnormal expression and activity of CCL5 and its receptor CCR5 have been found in hematological malignancies and solid tumors [20]. Elevated CCL5 levels have also been described in multiple myeloma [8]. CCL5 activity is mediated by binding to CCR5 and also to CCR1 and CCR3 receptors [14]. In multiple myeloma, blockage of the CCL5/receptor axis leads to inhibition of osteoclast formation and myeloma cell adhesion to stromal cells [21,22]. Cytokines and growth factors are produced and secreted by myeloma cells in the BM microenvironment and are regulated by autocrine and paracrine loops. Hence, the expressions of CCL5 can be regulated by the NF-κB factor after activation by other cells [23,24]. However, it has been proven that selected polymorphisms in the *CCL5* gene affect the level of its transcription. The human *CCL5* gene is located on chromosome 17 (*locus* 17q12) and consists of a promoter region, three exons, and two introns. The *CCL5* gene is short but filled with many polymorphisms, three of which, may affect *CCL5* expression: rs2107538 (g.−403G > A), rs2280788 (g.-28C > G), and rs2280789 (g.In1. + 1T > C) [25,26,27]. 

The *CCL5* variants have been associated with an increased risk of several cancers including: gastric cancer [28], prostate cancer [29], and breast cancer [30]. Moreover, correlations were found between the progression of pancreatic adenocarcinoma and colon cancer diseases and particular genotypes of the *CCL5* gene [19,31]. However, to date, there are no publications regarding the possible effect of the *CCL5* polymorphisms on MM risk and outcome.

Taking into account the above literature data, we hypothesize that *CCL5* and *CCR1* variants may be associated with the risk of MM development, and these variants may also affect patient response to treatment. The present study investigates the selected *CCL5* and *CCR1* variants, their association with selected clinical and laboratory disease parameters, and the response to bortezomib/thalidomide-based therapies. To our knowledge the presented results were not previously published by other authors.

## 2. Materials and Methods

### 2.1. Patients and Samples

A total of 201 unrelated subjects with high quality of DNA, comprising 101 newly diagnosed patients with MM and 100 health controls, were included in this study. Controls and samples were selected from the same ethnic group living in south-western Poland (Caucasian population). All MM patients were hospitalized between 2013 and 2019 at the Chair and Department of Hematooncology and Bone Marrow Transplantation, Medical University of Lublin. The study obtained positive opinions (no. KE-0254/165/2013 and no. KE-0254/337/2016) from the Bioethics Committee at the Medical University of Lublin, according with the ethical standards established by the Helsinki Declaration. All methods were performed in accordance with the relevant guidelines and regulations. Detailed patient characteristics are shown in Table 1. 

The healthy blood donors (50 males and 50 females, with mean age 37.6 years) visited the Regional Blood Donation and Blood Treatment Center in Kielce, Poland. All participants of the study provided written informed consent. The inclusion and exclusion criteria for all individuals included in the study are described in Table 2. 

Therapeutic induction regimens consisted of thalidomide and/or bortezomib combined with steroids and/or cyclophosphamide. The group of 37 MM patients underwent autologous hematopoietic stem cell transplantation (auto-HSCT). Response to treatment was evaluated according to the International Myeloma Working Group guidelines, as described elsewhere [32,33]. Overall survival (OS) encompassed time from diagnosis until relapse, progression, death due to tumor effect or last follow-up, and time from diagnosis until death by any cause or last follow-up, respectively. The median follow-up time of MM patients enrolled in the study was 18.16 months. Progression-free survival (PFS) was estimated as the time elapsed between treatment initiation and tumor progression or death from any cause [34].

Peripheral blood from healthy blood donors and bone marrow aspirates from MM patients were used for DNA isolation and determination of *CCL5* and *CCR1* variants. The samples of serum (*n* = 70) were used to determine the level of regulated on activation, normal T-cell expressed and secreted (RANTES)/chemokine (C-C motif) ligand (CCL5) in MM patients. 

Cell cultures were established from bone marrow aspirates to carry out the research associated with bortezomib treatment (*n* = 50), as described by Zmorzynski et al. [35]. 

### 2.2. DNA Isolation 

DNA isolation was performed using a commercial kit (Qiagen, Germany) according to the manufacturer’s procedure. The concentration and quality of DNA was checked using a NanoDrop device (Thermo Fisher Scientific, USA, Waltham, MA, USA). 

### 2.3. Genotyping

We selected the most analyzed genetic variants of *CLL5* (rs2280789, rs2280788, and rs2107538) and *CCR1* (rs3181077) genes. Genotyping was performed using TaqMan SNP genotyping assays on Applied Biosystems (USA, Waltham, MA, USA). For genotyping analysis, 7500 Fast Real-time PCR (Applied Biosystems, USA, Waltham, MA, USA) was used.

### 2.4. Enzyme-Linked Immunosorbent Assay (ELISA)

A specific ELISA kit (MyBioSource, San Diego, CA, USA) was used (according to manufacturer’s protocol) to determine the level of RANTES/CCL5 in serum samples collected from 70 MM patients. The plate reader (TK Biotech, Poland, Warsaw) at wavelength of 450 nm for measurement of RANTES/CCL5 was used. The serum samples were diluted 20 times. The concentration read from the standard curve was multiplied by the dilution factor (2×).

### 2.5. Bortezomib In Vitro Treatment 

**Figure 1:**
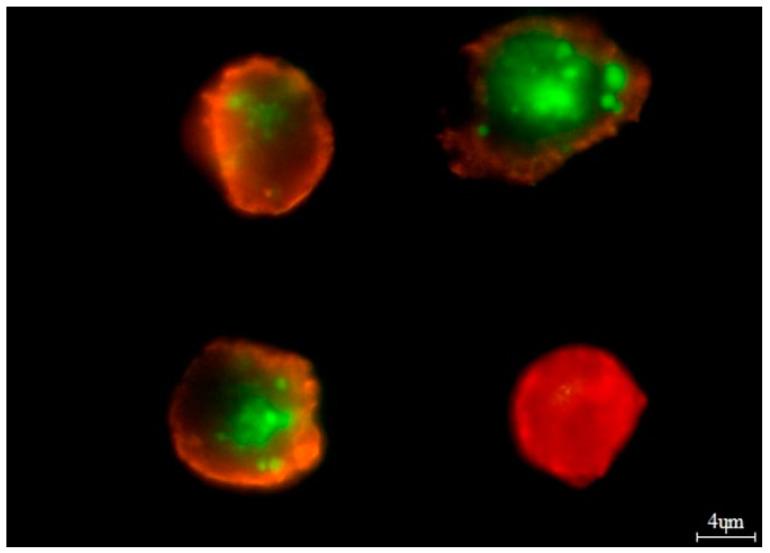
Example of *in vitro* bortezomib treatment. The viable cells were stained with 6-CF (6-carboxyfluorescein)—green dye. The necrotic cells were stained only with AnnCy3 (Annexin V Cy3.18)—red dye (cell in the lower right corner). The cells undergoing apoptosis were stained both with AnnCy3 (red) and 6-CF (green). In the figure, two cells on the left show late apoptosis. The cell in the upper right corner shows early apoptosis.

Bone marrow aspirates (*n* = 50) (mean number of plasma cells—31.31% ± 20.69) were used to establish cell cultures as described previously [35]. The number of apoptotic, necrotic, and viable cells was evaluated by means of the Annexin V-Cy3 Apoptosis Detection Kit according to manufacturer’s protocol (Sigma-Aldrich, USA, Saint Louis, MO, USA) (Figure 1). 

### 2.6. Statistical Analysis

Laboratory values of MM patients with studied variants were compared using an independent t-test for continuous variables and a Chi-square test for categorical variables. The association of studied variants with clinical data was evaluated using a Chi-square test or Fisher’s exact test (when one expected value was <5). The quantitative data was shown as frequency or percentage. Deviation of genotype frequencies in controls (healthy blood donors) and cases (MM patients) from Hardy–Weinberg equilibrium (HWE) was assessed by a Chi-square test with Yates’s correction for the groups with less than five patients [36]. For the 95% confidence interval (CI), we assumed *p* = 0.05 and χ^2^ = 3.84; therefore, if the χ^2^ ≤ 3.84 and the corresponding *p* ≥ 0.05, then the population is in HWE, as described previously [37]. The Cox proportional hazard model was used for univariate and multivariate analysis of OS and PFS. The Kaplan–Meier method and the log-rank test were used for survival analysis. Pairwise linkage disequilibrium (LD) was measured using D’ and Dmax values, as well as the squared correlation coefficient r^2^. Pearson’s correlation analysis was used to evaluate the correlation between RANTES/CCL5 concentration and laboratory/clinical data (free light chain ratio, age, % of plasma cells in bone marrow, number of platelets, estimated glomerular filtration rate concentration of: hemoglobin, albumins, β2-microglobulin, calcium ions, creatinine, and C-reactive protein). We assumed a 5% error of inference and the related level of significance *p* < 0.05, pointing to the existence of statistically significant differences. Statistical analyses were performed using the Statistica ver. 12.5 (StatSoft) software.

## 3. Results

The presented study included 101 MM patients (53 males and 48 females). The variants of *CCL5* and *CCR1* genes, as well as the level of RANTES/CCL5 in serum of MM patients were analyzed. Moreover, we performed cell cultures in a subgroup of MM patients (from bone marrow samples) with bortezomib to check whether the genotypes of *CCL5* and *CCR1* genes may be related to the effects of this drug.

### 3.1. Frequencies of Alleles and Genotypes and Their Association with MM Risk

Genotyping was successful in all the individuals investigated within the study. This was one of the inclusion criteria for MM patients and healthy blood donors. The *CCL5* and *CCR1* variants were in Hardy–Weinberg equilibrium (Table 3). 

Two variants—rs2280788 and rs2107538—were in the same haplotype block in MM patients and the control group—D’ = 0.90 and D’ = 0.91, respectively (Table 4). The correlation factor in all studied variants was low in MM patients and healthy blood donors—r^2^ range 0.18–0.31 and 0.09–0.25, respectively (Table 4).

The *CCL5* and *CCR1* variants were balanced (Table 5). We did not observe statistically significant differences between allele and genotype frequencies among MM patients and healthy blood donors. In the case of CT and CT + TT genotypes of rs2107538, as well as T-allele of rs2107538, statistical tendency was observed with the risk of MM development (Table 5). 

### 3.2. CCL5 and CCR1 Variants as a Risk Factors of Death and MM Progression

Minor genotypes were analyzed together with heterozygotes due to their small sample size. The only exception is for CC genotype of rs318077 variant due to there being a sufficient number of this rare/minor genotype—*n* = 9 (of MM patients) and *n* = 12 (of healthy blood donors). A univariate Cox analysis revealed that patients at stage III according to ISS had a 2.80-fold (*p* = 0.004) increased risk of death (Table 6). In the case of MM patients with auto-HSCT, lower risk of death was observed. Similar findings were observed in the case of disease relapse or progression in MM patients at stage III according to ISS (HR = 2.79, *p* < 0.001) and with auto-HSCT (HR = 0.39, *p* = 0.03) (Table 6). The univariate Cox analysis did not show the impact of analyzed variants on the risk of death or disease relapse or progression in MM patients. 

The multivariate Cox regression analysis confirmed that patients with auto-HSCT had a decreased risk of death and disease relapse or progression (Table 7). In contrast, patients at stage III according to ISS had an increased risk of death and disease relapse or progression. Moreover, multivariate Cox regression showed increased risk of disease relapse or progression (HR = 4.77; *p* = 0.01) in MM patients with CG + CC genotypes of *CCL5* rs2280788 variant. In the case of CT + TT genotypes of *CCL5* rs2107538 variant, decreased risk of death (HR = 0.18; *p* = 0.028) and disease relapse or progression were observed (HR = 0.26; *p* = 0.01) (Table 7). 

The analysis of response rate showed that MM patients without auto-HSCT had an increased chance of progressive disease (PD) (Table 8). Similar results were observed in MM patients with genotypes AG + GG (rs2280789) of *CCL5* gene (Table 8). 

### 3.3. Association of Studied Variants with Clinical/Laboratory Values

We analyzed potential relationships between clinical/laboratory results and selected appropriate genotypes. We found that AG + GG genotypes of *CCL5* rs2280789 were associated with lower levels of C-reactive protein. At the level of tendency, the changes in the concentration of creatinine (in rs2280789 variant), C-reactive protein (in rs2107538 variant), albumins (in rs318077 variant), % of plasma cells (in rs318077 variant), and estimated glomerular filtration rate (in rs318077 variant) were observed (Table 9). The CC + CT genotypes (of rs318077 variant) and CT + TT genotypes (of 2107538 variant) were associated with higher (OR = 2.72, *p* = 0.028) and lower (OR = 0.32, *p* = 0.027) risk of chromosomal aberrations presence, respectively. Other variants were not associated with the presence of chromosomal aberrations. Moreover, we did not observe statistically significant associations between studied variants and specific types of chromosomal aberrations—del(17p13.1) or t(4;14).

### 3.4. Survival of MM Patients Taking into Account Type of Tratment and Studied Variants

**Figure 2:**
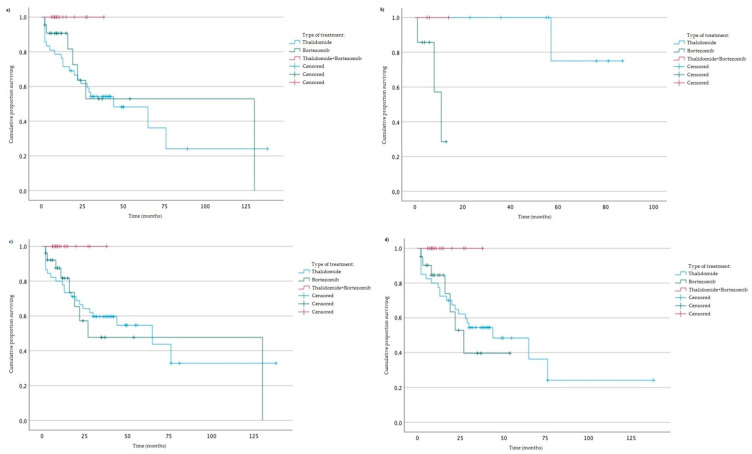
Kaplan-Meier analysis of OS taking into account the type of treatment: (**a**) AA genotype of rs2280789, log-rank test *p* = 0.026 (Thalidomide vs. Thalidomide + Bortezomib); (**b**) GA + GG genotypes rs2280789, log-rank test *p* = 0.008 (Thalidomide vs. Bortezomib); (**c**) GG genotype of rs2280788, log-rank test *p* = 0.025 (Thalidomide vs. Thalidomide + Bortezomib), *p* = 0.037 (Bortezomib vs. Thalidomide + Bortezomib), *p* = 0.051 (Thalidomide vs. Bortezomib); (**d**) CC genotype of rs2107538, log-rank test *p* = 0.025 (Thalidomide vs. Thalidomide + Bortezomib), *p* = 0.028 (Bortezomib vs. Thalidomide + Bortezomib).

We analyzed the association between studied genotypes and survival of MM patients (by log rank test). Without taking into account the type of treatment, we did not observe statistically significant changes in OS and PFS. Furthermore, a log rank (Mantel–Cox) analysis taking into account studied variants and the type of treatment (thalidomide vs. bortezomib vs. both thalidomide and bortezomib) was performed. We found an association of AA genotype of rs2280789 with the type of treatment and OS (*p* = 0.026) (Figure 2). Similar results were observed in the case of GA + GG genotypes of rs2280789 variant, GG genotype of rs2280788 variant, and CC genotype of rs2107538 variant (Figure 2). In MM patients with AA genotype (of rs2280789), GG genotype (of rs2280788) or CC genotype (of rs2107538), a higher survival rate in treatment with thalidomide and bortezomib was observed (Figure 2).

**Figure 3:**
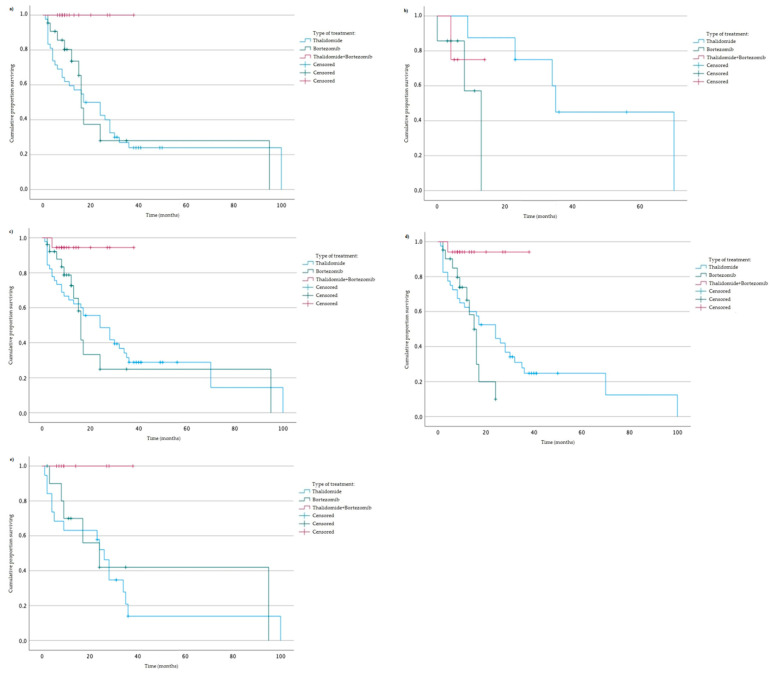
Kaplan–Meier analysis of OS taking into account the type of treatment: (**a**) AA genotype of rs2280789, log-rank test *p* = 0.002 (Thalidomide vs. Thalidomide + Bortezomib), *p* = 0.009 (Bortezomib vs. Thalidomide + Bortezomib); (**b**) GA + GG genotypes of rs2280789, log-rank test *p* = 0.022 (Thalidomide vs. Bortezomib); (**c**) GG genotype of rs2280788, log-rank test *p* = 0.01 (Thalidomide vs. Thalidomide + Bortezomib), *p* = 0.015 (Bortezomib vs. Thalidomide + Bortezomib); (**d**) CC genotype od rs2107538, log-rank test *p* = 0.008 (Thalidomide vs. Thalidomide + Bortezomib), *p* = 0.004 (Bortezomib vs. Thalidomide + Bortezomib); (**e**) TT genotypes of rs318077, log-rank test *p* = 0.015 (Thalidomide vs. Thalidomide + Bortezomib).

Moreover, in the analysis of treatment and PFS, we found statistically significant associations with AA genotype and GA + GG genotypes of rs2280789 variant, GG genotype of rs2280788 variant, CC genotype of rs2107538 variant and TT genotype of rs318077 variant (Figure 3). In MM patients with AA genotype (of rs2280789), GG genotype (of rs2280788), CC genotype (of 21107538) or TT genotype (of rs318077)—a higher PFS rate was found (Figure 3).

In the next step, we checked if there were differences in OS or PFS between major genotypes (of studied variants) and one type of treatment. There were no statistically significant differences between major genotypes and OS/PFS in patients treated with bortezomib (*p* = 0.905/*p* = 0.54), thalidomide (*p* = 0.826/*p* = 0.924) or both of these drugs (*p* = 0.392/*p* = 0.692), respectively.

### 3.5. Levels of RANTES/CCL5 in Serum of MM Patients

We investigated whether *CCL5* and *CCR1* variants have an impact on RANTES/CCL5 level. We found that their concentration depended on the type of studied genotype. Statistically significant lower RANTES/CCL5 levels were observed in minor genotypes and heterozygotes (Table 10). 

Moreover, we have analyzed the most frequent haplotypes and their association with RANTES/CCL5 concentration. Haplotypes with a frequency lower than 5% were excluded from our analysis. We observed a statistically significant difference in RANTES/CCL5 concentration between the two most common haplotypes (Table 11). All results obtained in a Pearson’s correlation analysis (RANTES/CCL5 concentration vs. clinical/laboratory data) were statistically insignificant, including C-reactive protein concentration (r = 0.038, *p* = 0.76) and % of plasma cells (r = 0.021, *p* = 0.86). 

### 3.6. Bortezomib In Vitro Treatment

**Figure 4:**
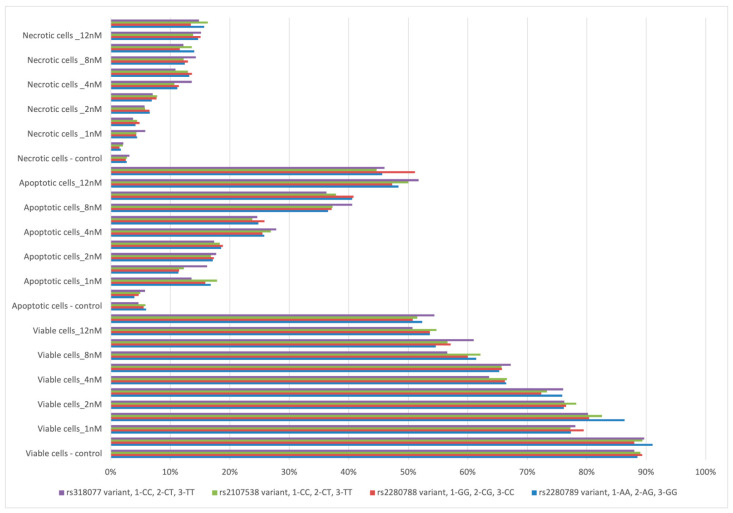
Bortezomib *in vitro* study taking into account *CCL5* and *CCR1* variants.

In *in vitro* studies, bortezomib increased the number of apoptotic and necrotic cells in all studied genotypes (Figure 4). However, in most cases the differences between the number of apoptotic, necrotic, or viable cells relative to bortezomib doses (for example, 1 nM vs. 2 nM) were statistically insignificant.

A higher number of apoptotic cells was observed at 1 nM of bortezomib in patients with AA genotype (of rs2280789) in comparison to those with GA + GG genotypes (16.79% vs. 11.37%, *p* = 0.021). A higher number of viable cells was found at 1 nM of bortezomib in cells with GA + GG genotypes (of rs2280789) in comparison to AA genotype—86.36% vs. 77.31%, *p* = 0.02.

## 4. Discussion

Multiple myeloma cells proliferate and grow mainly within bone marrow, where they create an environment that promotes disease progression, drug resistance, bone destruction, and immune escape [2]. MM and BM cells communicate with each other through the secretion and binding of cytokines and chemokines. The concentration of released chemokines may have a predictive value for the course of the disease. Reports published so far on the role of RANTES/CCL5 in cancer have been controversial. Some studies suggest that RANTES/CCL5 production may lead to a more immune-suppressive activity in tumor microenvironment (TME) [19], while other evidence suggests that RANTES/CCL5 is in favor of tumor immunity [40,41,42,43]. The expression, and thus the concentration of the RANTES/CCL5 chemokine, may be conditioned by external factors (stimulation by other cells) or genetic polymorphisms. Three single nucleotide polymorphisms (SNPs) in *CCL5*, namely rs2107538, rs2280788, and rs2280789, are the most frequent variants associated with inflammatory diseases. A growing body of research confirms that cytokine gene variants are an important factor in predicting the outcomes of the development and treatment of solid cancer diseases and hemato-oncological diseases. However, knowledge of *CCL5* gene polymorphisms implications with regard to MM susceptibility remains elusive. In our study, we analyzed the association of *CCL5* and *CCR1* variants with the risk and the outcome of MM as well as response to thalidomide and/or bortezomib treatment. In addition, we took into account the *in vitro* response to multiple doses of bortezomib.

In our research, we described association of the T-allele of rs2107538 with the risk of MM development at the level of tendency. We observed an increased risk of disease relapse or progression in MM patients with CG + CC genotypes rs2280788. A similar finding was observed in patients with TC + CC genotypes rs2280789. Our work is the first to examine the relationship between *CCL5* polymorphisms and the risk and course of MM disease. Several studies have found an association between *CCL5* variants and an increased risk of cancer. In the study by Shan et al., rs2107538-T allele was significantly associated with triple negative breast cancer (TNBC) [30]. Both *CCL5* rs2280788-GC and *CCL5* rs2280789-CC genotypes in their study showed a slightly significant association with TNBC risk [30]. Additionally, they showed that *CCL5* variants (rs2107538 and rs2280789) were linked to CCL5 serum and mRNA levels [30]. Eskandari-Nasab and colleagues demonstrated that *CCL5* rs2107538 variants were associated with an increased risk of breast cancer [44]. Their results indicated that individuals carrying the *CCL5* rs2107538-GA or GA + AA genotypes or A allele had higher risk of developing breast cancer compared to those carrying the CC genotype or C allele [44]. In Suenaga et al., they demonstrated that patients with colorectal cancer who possessed *CCL5* rs2280789 G alleles had poorer outcomes with shortened PFS and OS rates, as well as poor tumor response compared to those with the AA variant. Interestingly, they also found that CCL5 was not only expressed in cancerous tissue, but also in non-neoplastic mucosal tissues, and the clinical impact of this *CCL5* allele did not differ depending on primary tumor location in the colon [19].

There are no studies on the involvement of *CCL5* polymorphisms in the development of hematological malignancies. However, several studies have been published on the association of *CCL5* polymorphic variants with the development of graft-versus-host (GVHD) disease in hematological patients after allo-HSCT. The development of GVHD involves soluble and cellular components of both the adaptive and innate immune response. The migration of leukocytes to and from secondary lymphoid tissues is therefore an essential component of GVHD [45,46]. Data from Choi et al., suggest that CCR1/CCL5 receptor–ligand interactions play a role in allo-specific T-cell responses and demonstrate that CCR1 expression on donor cells contributes to the development of GVHD [47]. Kim and colleagues showed that the CG genotype of rs2280788 in recipients of allo-HSCT was significantly associated with a higher incidence of chronic GVHD, extensive chronic GVHD, and severe grade of chronic GVHD compared to CC genotype [48]. In contrast, a study by Shin et al. suggested that *CCL5* variants may be associated with acute GVHD rather than chronic GVHD, as well as relapse-free survival in patients treated with allo-HSCT [49]. In the studies presented above, *CCL5* polymorphisms have been rather negative prognostic factors of the risk of developing graft versus host disease. In our study, MM patients with auto-HSCT were included, and not those with allo-HSCT. Moreover, a lower risk of death and disease relapse or progression in MM patients with auto-HSCT was observed.

Several studies have found an association between *CCL5* polymorphisms and decreased risk of cancer and other diseases. Liou and colleagues indicated that women who inherit A allele of *CCL5* rs2107538 may be at reduced risk of gastric cancer [28]. A study by Singh et al. suggests that TT genotype of the *CCL5* rs2280789 variant plays an important role in increased CCL5 expression in T cells, which may enhance Th1 immunity and help in protection against tuberculosis [50]. This suggests that increased CCL5 expression strengthens the defensive properties of the immune system. Qiu et al. through their work showed that CCL5 can play an anti-tumor role in breast cancer [51]. CCL5/RANTES secretion was induced by IL-27 activity. In the presented materialthe presence of the T-allele of *CCL5* rs2107538 variant was associated with higher risk of MM at the level of tendency.

In our study we described some positive impacts of the studied variants on the course of the MM disease. We found higher PFS rates in individuals with CC-rs2107538, GG-rs2280788, and AA-rs2280789. Additionally, the same individuals were in the group with higher survival rate in treatment with thalidomide and bortezomib. In the case of GA + GG genotypes of rs2280789 variant poor response to bortezomib was observed. It should be noted that GA + GG genotypes were associated with statistically lower CCL5/RANTES level in comparison to AA genotype (of rs2280789). The best response to treatment should be observed in patients being treated with both—bortezomib and thalidomide. These drugs are characterized by different modes of action. Immune dysfunction including dendritic cells deficiencies is a hallmark of MM. These cells play an important role in MM pathophysiology. The lack of their proper immunological function results in drug resistance and the subsequent failure of immunotherapeutic approaches [52]. Dendritic cells that were exposed to bortezomib showed reduced secretion of chemoattractants involved in inflammation and lymphocyte recruitment such as CCL5/RANTES [53,54]. Kuwahara-Ota and colleagues found that secretion of CCL5/RANTES by myeloma cells is a prerequisite for induction of immunosuppressive myeloid-derived suppressor cells in MM [55]. An increase in immunosuppressive myeloid-derived suppressor cells is associated with MM progression and treatment resistance. Thalidomide has been shown to possess immunomodulatory attributes, including the inhibition of cytokine production including CCL5/RANTES [56]. A new class of thalidomide derivatives was developed. These immunomodulatory drugs (IMiDs) are structurally related and have their unique set of anti-inflammatory, immunomodulatory, antiproliferative, antiangiogenic and toxicity profiles [57]. ImiDs were identified as potent inhibitors of immunosuppressive myeloid-derived suppressor cells induction through independent downregulation of CCL5/RANTES in myeloma cells, and downregulation of a receptor for CCL5 chemokine [55]. The poor response to bortezomib in comarison to thalidomide exhibited in the GA-GG genotypes may be due to stronger effect of thalidomide (via inhibition of immunosuppressive myeloid-derived suppressor cells).

We analyzed CCL5/RANTES concentration in the serum of MM patients and its correlation with clinical/laboratory data. No statistically significant correlations were found. Nevertheless, we found that CCL5/RANTES concentration depended on the type of studied genotype of *CCL5* and *CCR1* gene. Lower concentration of CCL5/RANTES were observed in a group of patients with minor genotypes and heterozygotes (analyzed together as one group) of all studied variants. Based on these results, it can be concluded that individual genotype and haplotype affect the expression and secretion of the CCL5/RANTES in MM patients. In addition to neoplastic diseases, *CCL5* variants may be a positive factor in the course of other inflammatory and/or autoimmune diseases. Van Veen and colleagues found that the low-producer *CCL5* allele rs2107538-G was associated with reduced risk of severe axonal loss, whereas the high-producer *CCL5* allele rs2107538-A was associated with a worse clinical course of multiple sclerosis [58]. In a study by Zhernakova et al., *CCL5* variants were significantly associated with serum concentration of chemokine and development of diabetes type 1 (T1D) [59]. The rs4251719*A-rs2306630*A-rs2107538*A haplotype was associated with low CCL5/RANTES production and confers protection from T1D [59].

The number of studies related to the role of *CCR1* variants in neoplastic diseases is very limited. CCR1 is involved in the recruitment of inflammatory immune cells including neutrophils, monocytes, and lymphocytes [60]. CCL5/RANTES activity is mediated by binding to CCR1 and also to CCR5 receptors [14]. In our studies, we included only one variant of the *CCR1* gene—rs318077. We found that individuals with the TT genotype of rs318077 had higher PFS rates. Further CT + TT genotypes of rs318077 were associated with higher risk of chromosomal aberrations.

There are some limitations of our study. The number of MM patients was relatively small, in part due to the low incidence of the disease. However, the group of MM patients included in the study was large enough for most analyses. Some analyses were not possible as a result of low frequency of alleles in the population. For evaluation of apoptosis in our *in vitro* study, fluorescent microscopy was applied instead of flow cytometry-based apoptosis detection (FACS). FACS analysis is more reliable than quantitative apoptosis evaluation. In addition, the FACS method differentiates between cells in early and late apoptosis. Unfortunately, retrospective analysis of apoptosis with the use of flow cytometry is not possible. The set used for apoptosis and necrosis detection was dedicated and validated to fluorescent microscopy. Analysis of CCL5/RANTES level was performed in serum. Using bone marrow plasma instead of serum would have been more informative.

Despite this study’s limitations and the need for prospective studies with larger sample sizes, our findings suggest that major genotypes of *CCL5* variants may be independent positive prognostic factors in MM.

## 5. Conclusions

In conclusion, the results of this study suggest that *CCL5* variants may have positive prognostic implications for MM. Moreover, our results show that *CCL5* variants may be predictors of thalidomide and bortezomib treatment response in MM. The presence of major genotypes of rs2280789, rs2280788, and rs21107538 was associated with higher OS and PFS.

## Figures and Tables

**Table 1:** The characteristics of MM patients included in the study.

Variables	MM Patients, *n* = 101
Age (years)	65.46
Sex	
Male	53
Female	48
Type of MM *	
IgG	56
IgA	26
Light chain	19
Stage according to the International Staging System *	
I	28
II	30
III	43
Smoking	
Yes	20
No: Non-smokers	69
No: Ex-smokers	12
Exposure to carcinogenic factors	
Yes	28
No	73
Renal failure *	
No	82
Yes	19
The stage of chronic kidney disease (grade)	
G1	30
G2	28
G3A	16
G3B	12
G4	7
G5	8
Anemia grade before treatment (WHO)	
Absent	28
I—mild	33
II—moderate	30
III—severe	10
Cytogenetic changes *	
del(17p13.1)	14
t(4;14)	15
Other types	2
Chemotherapy	
Cyclophosphamide, Thalidomide, and Dexamethasone (CTD)	50
Velcade, Cyclophosphamide, Dexamethasone (VCD)	29
Velcade, Thalidomide, and Dexamethasone (VTD)	20
Died before chemotherapy	2

* at diagnosis.

**Table 2:** Inclusion and exclusion criteria for MM patients and blood donors.

Inclusion Criteria for MM Patients
-Newly diagnosed MM patients. -Signed informed consent -18 years of age or older -Measurable disease, defined as follows: For secretory MM—the presence of quantifiable monoclonal component, ≥0.5 g/dL For poor secretory or non-secretory MM—the level of the affected serum free light chain must be ≥ 10 mg/dL (≥100 mg/L, with an abnormal free light-chain ratio) -Eastern Cooperative Oncology Group (ECOG) Performance status ≤3 -Life expectancy more than 3 months -Successful genotyping
Inclusion criteria for control group
-18 years of age or older -Signed informed consent -Successful genotyping
Exclusion criteria for MM patients
-Active smoldering MM -Active plasma cell leukemia -Documented systemic amyloid light chain amyloidosis -Active central nervous system involvement with MM -Other active hematologic malignancy or solid tumor
Exclusion criteria for Control group
-Known to be infected with HIV, syphilis, tuberculosis, hepatitis B, or hepatitis C -A condition in which repeated blood draws or injections pose more than minimal risk for the subject such as hemophilia, other severe coagulation disorders, or significantly impaired venous access -A condition that requires active medical intervention or monitoring to avert serious danger to the participant’s health or well-being

**Table 3:** Hardy–Weinberg equilibrium (HWE) for *CCL5* and *CCR1* variants in the case and control groups according to expected (E) and observed (O) values.

GROUPS	GENOTYPES			Total	HWE *p* Value and χ^2^ *
*CCL5* gene rs2280789					
-	AA	AG	GG	-	-
CONTROL					
E	85.56	13.87	0.56	100	*p* = 0.51, χ^2^ = 0.42
O	86	13	1	100	
CASE					
E	80.19	19.6	1.19	101	*p* = 0.09 χ^2^ = 2.84
O	82	16	3	101	
*CCL5* gene rs2280788					
-	GG	CG	CC	-	-
CONTROL					
E	89.3	10.39	0.3	100	*p* = 0.18, χ^2^ = 1.73
O	90	9	1	100	
CASE					
E	91.24	9.5	0.24	101	*p* = 0.55, χ^2^ = 0.34
O	91	10	0	101	
*CCL5* gene rs2107538					
-	CC	CT	TT	-	-
CONTROL					
E	66.42	30.15	3.42	100	*p* = 0.71, χ^2^ = 0.13
O	67	29	4	100	
CASE					
E	78.42	21.14	1.42	101	*p* = 0.17, χ^2^ = 1.83
O	80	18	3	101	
*CCR1* gene rs318077					
-	TT	CT	CC	-	-
CONTROL					
E	44.89	44.22	10.89	100	*p* = 0.68, χ^2^ = 0.16
O	46	42	12	100	
CASE					
E	43.12	45.74	12.12	101	*p* = 0.3, χ^2^ = 1.05
O	40	52	9	101	

* if the χ^2^ ≤ 3.84 and the corresponding *p* ≥ 0.05 then the population is in HWE.

**Table 4:** Pairwise linkage disequilibrium and squared correlation coefficient r^2^ taking into account *CCL5* variants in MM patients and healthy blood donors.

*CCL5* Variants	Individuals	D Value	Dmax Value	D’Value	r^2^ Value
rs2280789 and rs2280788	MM patients	0.026	0.045	0.57	0.18
	Control group	0.023	0.036	0.64	0.25
rs2280789 and rs2107538	MM patients	0.050	0.090	0.55	0.31
	Control group	0.035	0.065	0.54	0.09
rs2280788 and rs2107538	MM patients	0.040	0.044	0.90	0.20
	Control group	0.029	0.032	0.91	0.13

**Table 5:** The comparison of allele frequency and distribution of *CCL5* and *CCR1* variants among MM patients and controls.

Gene Variants and Alleles	MM *n* (%)	Controls *n* (%)	Odds Ratio	95% CI	*p* Values
*CCL5* rs2280789					
AA	82 (81.18%)	86 (86%)	1	-	-
AG	16 (15.84%)	13 (13%)	0.77	0.35–1.71	0.52
GG	3 (2.97%)	1 (1%)	3.14	0.32–30.86	0.59
AG + GG	19 (18.81%)	14 (14%)	0.70	0.33–1.49	0.35
Total:	101 (100%)	100 (100%)			
A	180 (89.1%)	185 (92.5%)	1	-	-
G	22 (10.9%)	15 (7.5%)	0.66	0.33–1.31	0.24
Total:	202 (100%)	200 (100%)			
*CCL5* rs2280788					
GG	91 (90.1%)	90 (90%)	1	-	-
CG	10 (9.9%)	9 (9%)	0.91	0.35–2.34	0.84
CC *	0 (0%)	1 (1%)			
CG + CC	10 (9.9%)	10 (10%)	1.01	0.40–2.54	1.0
Total:	101 (100%)	100 (100%)			
G	192 (95%)	193 (96,5%)	1	-	-
C	10 (5%)	7 (3.5%)	0.69	0.25–1.86	0.47
Total:	202 (100%)	200 (100%)			
*CCL5* rs2107538					
CC	80 (79.2%)	67 (67%)	1	-	-
CT	18 (17.8%)	29 (29%)	1.92	0.98–3.76	0.05
TT	3 (2.97%)	4 (4%)	1.59	0.34–7.36	0.83
CT + TT	21 (20.77%)	33 (33%)	1.87	0.99–3.64	0.05
Total:	101 (100%)	100 (100%)			
C	178 (88.1%)	163 (81.5%)	1	-	-
T	24 (11.8%)	37 (18.5%)	1.68	0.96–2.93	0.06
Total:	202 (100%)	200 (100%)			
*CCR1* rs318077					
TT	40 (39.6%)	46 (46%)	1	-	-
CT	52 (51.5%)	42 (42%)	0.70	0.39–1.26	0.23
CC	9 (8.9%)	12 (12%)	1.16	0.44–3.03	0.76
CT + CC	61 (60.4%)	54 (54%)	0.76	0.43–1.34	0.35
Total:	101 (100%)	100 (100%)			
T	131 (65%)	134 (67%)	1	-	-
C	71 (35%)	66 (33%)	0.90	0.60–1.37	0.64
Total:	202 (100%)	200 (100%)			

* too small group for analysis.

**Table 6:** Univariate Cox analysis in survival of MM patients.

Variable	Univariate Cox Analysis for OS			Univariate Cox Analysis for PFS		
	*p* Value	HR	95% CI	*p* Value	HR	95% CI
ISS						
I + II	-	R		-	R	-
III	0.004	2.78	1.40–5.38	<0.001	2.79	1.61–4.84
Auto-HSCT						
yes	<0.001	0.18	0.07–0.46	0.03	0.39	0.21–0.72
no	-	R	-	-	R	-
*CCL5* rs2280789						
AA	-	R	-	-	R	-
AG + GG	0.21	0.51	0.17–1.43	0.73	0.88	0.43–1.81
*CCL5* rs2280788						
GG	-	R	-	-	R	-
CG + CC	0.64	0.75	0.22–2.50	0.31	1.61	0.63–4.12
*CCL5* rs2107538						
CC	-	R	-	-	R	-
CT + TT	0.12	0.46	0.14–1.20	0.17	0.59	0.27–1.26
*CCR1* rs318077						
CC	-	R	-	-	R	-
CT + TT	0.90	0.98	0.70–1.38	0.58	0.92	0.70–1.22

R-reference.

**Table 7:** Multivariate Cox analysis in survival of MM patients.

Variable	Multivariate Cox Analysis for OS			Multivariate Cox Analysis for PFS		
	*p* Value	HR	95% CI	*p* Value	HR	95% CI
ISS						
I + II	-	R	-	-	R	-
III	0.049	2.16	1.0–4.66	0.001	2.80	1.50–5.20
Auto-HSCT						
yes	-	R	-	-	R	-
no	0.03	0.19	0.07–0.56	0.046	0.49	0.24–0.99
*CCL5* rs2280789						
AA	-	R	-	-	R	-
AG + GG	0.74	0.80	0.20–3.17	0.82	1.10	0.45–2.70
*CCL5* rs2280788						
GG	-	R	-	-	R	-
CG + CC	0.15	3.77	0.61–23.34	0.01	4.77	1.42–15.99
*CCL5* rs2107538						
CC	-	R	-	-	R	-
CT + TT	0.028	0.18	0.04–0.83	0.01	0.26	0.09–0.73
*CCR1* rs318077						
CC	-	R	-	-	R	-
CT + TT	0.99	0.99	0.70–1.44	0.41	0.88	0.65–1.20

R-reference.

**Table 8:** *CCL5* and *CCR1* variants in response rate of MM patients.

Variable	Response Rate	
	CR + VGPR + PR + SD	PD
	*p* Value	OR (95% CI)
ISS		
I + II	-	reference
III	0.07	3.06 (0.65–14.39)
Auto-HSCT		
yes	-	reference
no	0.02	4.02 (1.26–12.87)
*CCL5* rs2280789		
AA	-	reference
AG + GG	0.01	1.10 (0.35–3.45)
*CCL5* rs2280788		
GG	-	reference
CG + CC	0.41	1.17 (0.28–4.82)
*CCL5* rs2107538		
CC	-	reference
CT + TT	0.92	0.93 (0.30–2.88)
*CCR1* rs318077		
TT	-	reference
CT + CC	0.86	0.92 (0.36–2.33)

CR-complete response, VGPR-very good partial response, PR-partial response, SD-stable disease, PD-progressive disease patients—according to response criteria for MM [38,39].

**Table 9:** The clinical values of MM patients included to the study taking into account studied variants.

Variables	MM Patients	*CCL5* rs2280789			*CCL5* rs2280788			*CCL5* r rs2107538			*CCR1* rs318077		
		AA	AG + GG	*p*-Value	GG	CG + CC	*p*-Value	CC	CT + TT	*p*-Value	TT	CT+ CC	*p*-Value
Mean age (years) *	65.46	65.80	64.0	0.48	65.84	62.10	0.26	65.72	64.48	0.61	62.20	65.64	0.83
Free light chain ratio *	303.05	332.9	178.6	0.44	309.3	248.0	0.81	302.9	303.7	0.98	433.0	224.2	0.20
% of plasma cells in bone marrow *	30.80	31.28	37.32	0.44	30.73	28.70	0.76	29.97	32.62	0.58	35.07	27.62	0.07
Albumins (g/dL) *	3.57	3.55	3.64	0.62	3.58	3.45	0.56	3.57	3.56	0.97	3.72	3.46	0.05
β2-microglobulin * (mg/L)	6.12	6.06	6.37	0.77	6.02	7.05	0.46	5.73	7.55	0.17	6.37	5.96	0.65
Calcium * (mM/L)	2.45	2.45	2.44	0.96	2.46	2.37	0.41	2.46	2.41	0.54	2.40	2.48	0.21
Hemoglobin * (g/dL)	10.40	10.41	10.33	0.87	10.45	9.90	0.38	10.44	10.21	0.62	10.06	10.61	0.15
Creatinine * (mg/dL)	1.60	1.67	1.15	0.05	1.60	1.31	0.60	1.58	1.54	0.93	1.74	1.47	0.43
Platelets (K/μL)	212.75	214.2	206.3	0.74	216.0	183.1	0.29	217.4	195.0	0.32	198.5	222.1	0.21
C-reactive protein * (mg/L)	15.53	17.54	6.56	0.02	16.54	6.11	0.39	18.47	4.82	0.05	10.50	18.86	0.25
Estimated glomerular filtration rate * mL/min/1.73 m^2^	60.92	65.84	70.19	0.58	66.51	68.02	0.88	66.97	65.47	0.84	56.24	73.50	0.05

* at diagnosis.

**Table 10:** Changes in the concentration of RANTES/CCL5 in serum samples from MM patients.

Gene Variant	Genotypes	Number of Individuals	Mean Concentration (ng/mL)	Standard Deviation	*p* Value
rs2280789	AA	56	11.15	1.37	<0.01
	GA + GG	14	6.43	3.20	
rs2280788	GG	64	10.80	2.10	<0.01
	CG + CC	6	3.85	3.44	
rs2107538	CC	56	10.95	1.76	<0.01
	CT + TT	14	7.22	3.70	
rs3181077	TT	33	11.27	3.05	0.014
	CT + CC	37	9.25	2.99	

**Table 11:** Haplotypes comprising *CCL5* and *CCR1* variants and concentration of RANTES/CCL5 (ng/mL).

*CCL5* and *CCR1* Variants				Frequency	Mean Concentration (ng/mL)	Standard Deviation	*p* Value
rs2280789	rs2280788	rs2107538	rs3181077				
AA	GG	CC	TT	0.37	11.73	1.25	0.03
AA	GG	CC	CT	0.30	10.96	1.13	

## Data Availability

The clinical data used to support the findings of this study are available from the corresponding author upon request.
